# 2402: Long-term stability of cortical language sites following resective epilepsy surgery

**DOI:** 10.1017/cts.2017.270

**Published:** 2018-05-10

**Authors:** Robert Matthew Gramer, Sandra Serafini, David Madigan, Gerald Grant, George Ojemann

**Affiliations:** 1 School of Medicine, Duke University, Durham, NC, USA;; 2 Department of Neurosurgery, Duke University, Durham, NC, USA;; 3 Department of Statistics, Columbia University, New York, NY, USA;; 4 Department of Neurosurgery, Stanford University, Stanford, NY, USA;; 5 Department of Neurosurgery, University of Washington, Seattle, WA, USA

## Abstract

BACKGROUND: Of the ~50 million cases of epilepsy worldwide, an estimated 80% originate from cortical areas implicated in language. Although the precise language loci can vary significantly across individuals, electrical stimulation mapping for eloquent areas has become standard of care in resective surgery for frontotemporal epilepsies. Although considerable work has been done to establish the minimum necessary resection distance from these sites to preserve language, no previous work has shown how these representations are affected by proximal resections. METHODS: Between 1967 and 2005, 22 patients [seizure onset (y): 11.5 (0.2–33); age at initial resection (y): 27.7 (10–39); time between operations (y): 8.4 (1–20.3); sex: 14 females; hemisphere: 21 left] underwent repeated perisylvian resective epilepsy surgeries of the language-dominant hemisphere. Each set of operations comprised intraoperative language mapping and cortical photographs. Using this data, a Bayesian hierarchical model was used to estimate the variability of language localization pre-resection Versus post-resection. RESULTS: The statistical model shows the posterior median difference in cortical location of language sites preresection Versus postresection is 0.6 cm, with a posterior 95% CI of 0.4 cm, 0.9 cm. CONCLUSION: This work suggests permanence in cortical language centers following resection of infringing cortex, while providing a reasonable statistical method to impute unobserved sites during the mappings, and confirming the validity of using proximity sites defined by shortest distance in the current literature.

